# Barriers and facilitators to implement longstanding exercise therapy for people with rheumatoid arthritis or axial spondyloarthritis

**DOI:** 10.1093/rap/rkag013

**Published:** 2026-01-29

**Authors:** Annabelle R Iken, Maaike G J Gademan, Thea P M Vliet Vlieland, Aukje Nutma, Floris A van Gaalen, Astrid M van Tubergen, Rudolf W Poolman, Leti van Bodegom-Vos, L-EXTRA and L-EXSPA Implementation Study Group, W H van der Laan, S de Klerk, Maaike W F H Peter, P Pennings, C Rozema

**Affiliations:** Department of Orthopaedics, Rehabilitation, and Physical Therapy, Leiden University Medical Center, Leiden, The Netherlands; Department of Orthopaedics, Rehabilitation, and Physical Therapy, Leiden University Medical Center, Leiden, The Netherlands; Department of Clinical Epidemiology, Leiden University Medical Center, Leiden, The Netherlands; Department of Orthopaedics, Rehabilitation, and Physical Therapy, Leiden University Medical Center, Leiden, The Netherlands; Department of Innovation, Quality & Research, Basalt Rehabilitation, The Hague, The Netherlands; Department of Orthopaedics, Rehabilitation, and Physical Therapy, Leiden University Medical Center, Leiden, The Netherlands; Department of Rheumatology, Leiden University Medical Center, Leiden, The Netherlands; Department of Rheumatology, Maastricht University Medical Center, Maastricht, The Netherlands; Department of Orthopaedics, Rehabilitation, and Physical Therapy, Leiden University Medical Center, Leiden, The Netherlands; Department of Orthopaedic Surgery, Joint Research, Onze Lieve Vrouwe Gasthuis, Amsterdam, The Netherlands; Department of Biomedical Data Sciences – Medical Decision Making, Leiden University Medical Center, Leiden, The Netherlands

**Keywords:** rheumatoid arthritis, axial spondyloarthritis, qualitative research, implementation science, semi-structured interviews, physiotherapy

## Abstract

**Objectives:**

Longstanding, personalized, supervised exercise therapy proved to be (cost)effective for people with rheumatoid arthritis (RA) or axial spondyloarthritis (axSpA) with severe functional limitations. Despite policy support, implementation in routine care is challenging. This study aimed to identify barriers and facilitators to its uptake in clinical practice.

**Methods:**

We conducted 18 semi-structured interviews with key stakeholders (patients, rheumatologists, physiotherapists and insurers). The Consolidated Framework for Implementation Research (CFIR) guided the interviews. Interview transcripts were analyzed in Atlas.ti using direct content analysis, and findings were mapped to the CFIR domains. Barriers and facilitators were categorized across three healthcare delivery stages: (self)referral, eligibility assessment and treatment.

**Results:**

Barriers and facilitators were identified across all stages. Patients primarily mentioned adherence-related factors; rheumatologists focused on referral pathways; physiotherapists emphasized eligibility assessment and actual provision and insurers highlighted the extent of use and financial coverage. Cross-cutting barriers included eligibility criteria, limited access to trained physiotherapists, unclear referral processes and financial uncertainties. Facilitators included strong evidence of effectiveness, consistent messaging, clear information channels and availability of a training course for physiotherapists to deliver the longstanding exercise therapy.

**Conclusion:**

Despite policy support, implementing longstanding exercise therapy can be challenging across multiple stakeholder groups and healthcare delivery stages. A coordinated, multi-stakeholder approach is essential to address barriers while utilizing facilitators. Implementation strategies must improve referral processes, clarify eligibility criteria, enhance patient education and ensure the availability of trained physiotherapists.

Key messages

- Despite policy support and strong evidence, the implementation of (cost-)effective exercise therapy can be challenging.
- Focus on clarifying eligibility criteria, improving referrals and ensuring access to qualified physiotherapists and information.
- A coordinated, multi-stakeholder approach with shared responsibilities is crucial for successful implementation.

## Introduction

Rheumatoid arthritis (RA) and axial spondyloarthritis (axSpA) are chronic inflammatory diseases affecting approximately 0.46% and 0.20% of the global population, respectively [1, 2]. Although management of RA [3] and axSpA [4], particularly with effective biological agents, has advanced, many patients still experience severe functional disability and impaired health-related quality of life (HRQOL) [5]. This is often due to irreversible joint damage, disease complexity or other health conditions [6, 7].

**Figure 1:**
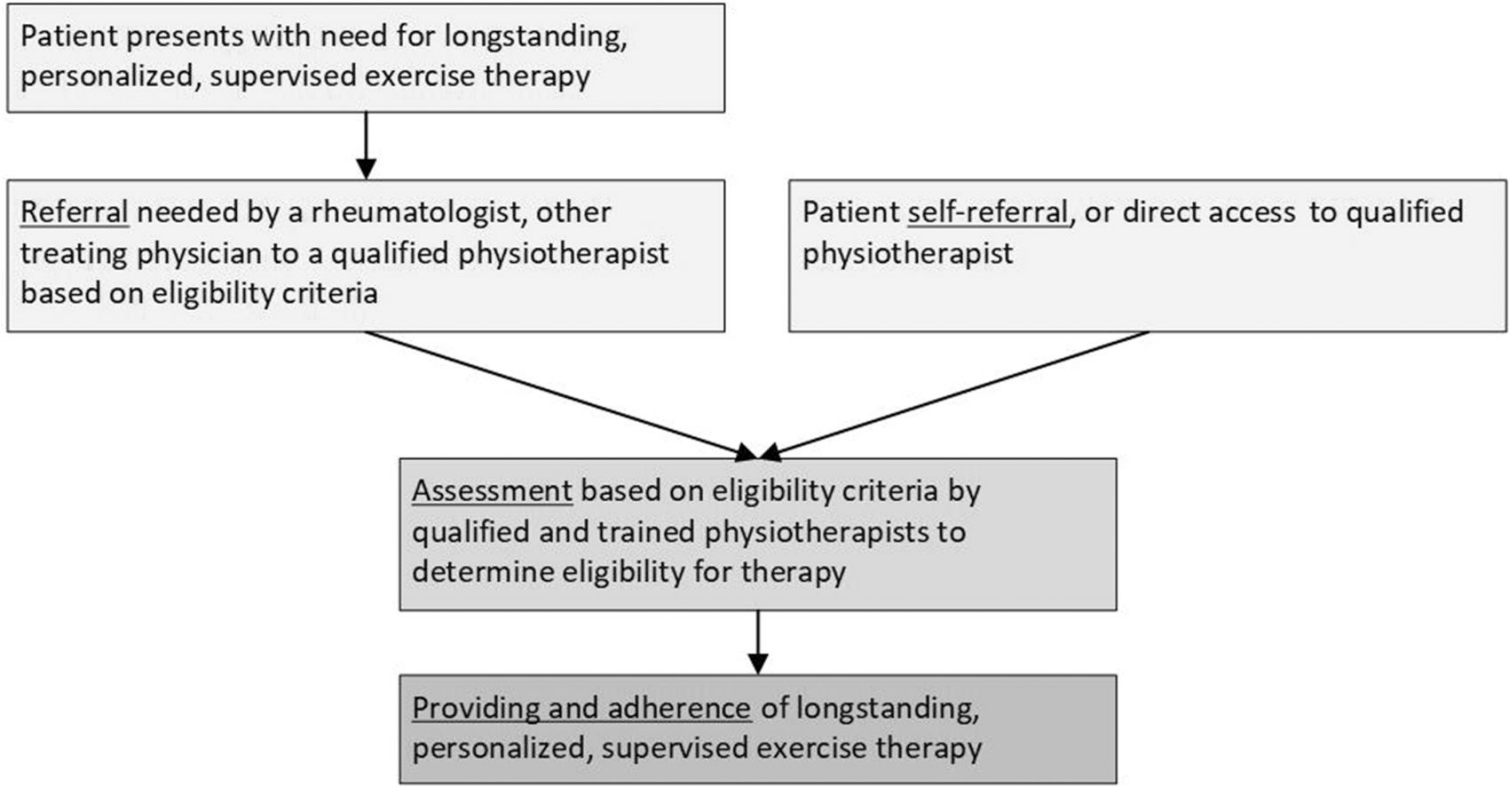
The three healthcare delivery stages of longstanding, personalized, supervised exercise therapy

Two recent Dutch randomized controlled trials demonstrated that longstanding exercise therapy delivered by trained physiotherapists is (cost-)effective for improving function and quality of life in people with RA or axSpA and severe functional limitations [8, 9]. Longstanding exercise therapy refers to a structured, supervised and personalized exercise program of at least 12 months’ duration, delivered by trained physiotherapists, to patients with RA or axSpA experiencing severe functional limitations (see Box 1). Based on these findings, Dutch basic insurance has resumed coverage for longstanding therapy after a period of non-reimbursement. In the Dutch healthcare system, implementing this therapy involves three healthcare delivery stages: (1) referral or direct access to longstanding exercise therapy, (2) patient eligibility assessment and (3) provision of patient treatment (Fig. 1).

Successfully navigating this multi-stage implementation process, however, may be challenging despite the evidence of the therapy’s (cost-)effectiveness and endorsements. The primary difficulty is that its delivery requires coordination between referring clinicians and treatment providers across organizational boundaries [10–16]. Given the context-dependent, multi-organizational nature of this intervention, a thorough understanding of both barriers and facilitators is crucial for optimizing implementation and improving patient outcomes.

While existing literature has identified important barriers within individual settings, such as patient motivation, physiotherapist skills, financial resources, funding and organizational support within physiotherapy [16–24], less attention has been paid to inter-organizational challenges that occur across the different stages of care. The aim of this study is to identify barriers and facilitators to the implementation of longstanding exercise therapy and uptake in clinical practice, from the perspectives of all stakeholders involved in its delivery. The findings from this analysis can then be used to inform the development of strategies for improving patient access to this care.

## Methods

### Setting

This study took place in the Dutch healthcare context, where patients can access physiotherapy directly but must typically pay for it themselves as it is not covered by basic insurance. The intervention studied was a longstanding (≥12 months), personalized, active exercise program targeting patients with severe functional disability and impaired quality of life (see Box 1 for additional information).

### Study design

A qualitative study employing semi-structured in-depth interviews was conducted between December 2023 and June 2024 to explore barriers and facilitators to implementing longstanding exercise therapy for patients with RA or axSpA and severe functional limitations. We used semi-structured interviews to capture diverse perspectives across (self-)referral, eligibility assessment and treatment [17]. The reporting of this study adheres to the COREQ (consolidated criteria for reporting qualitative research) guidelines for qualitative research [18].

### Participants

To understand the multi-level implementation process, we invited key informants involved in all stages of care: patients with RA or axSpA with significant disability were recruited via prior studies and the national patient organization (the National Association ReumaZorg Nederland (RZN)). Furthermore, the study included rheumatologists from various hospitals, physiotherapists from private practices across the Netherlands and health insurers who were recruited from a working group within the national organization, Zorgverzekeraars Nederland (ZN). A purposive and balanced sampling strategy was employed; if an individual declined, another with a similar background was asked to maintain a stratified balance across key characteristics, including diagnosis, sex and professional experience [17]. Following an email invitation and phone call, all participants provided written informed consent, which was verbally reconfirmed before the interview.

### In-depth interviews

We used the CFIR because it provides a comprehensive taxonomy of determinants across multiple levels (intervention, individuals, the inner and outer setting), which aligned with our aim to identify and organize barriers and facilitators across organizations and stakeholder groups. While other determinant frameworks (e.g. Theoretical Domains Framework (TDF), Tailored Implementation for Chronic Diseases (TICD) checklist) offer valuable perspectives, they capture fewer contextual levels or are developed for more specific purposes (e.g. individual behaviour or clinical guidelines). CFIR’s broad applicability across diverse implementation settings supported its suitability for our study [19, 20].

1. Intervention Characteristics—explores the features of the intervention itself, including its perceived advantage over existing practices, complexity, compatibility with current workflows and values, adaptability to local context, and costs.
2. Outer Setting—refers to the broader external environment that impacts an organization. This refers to the external environment in which the organization operates, encompassing factors beyond its direct control that can influence its functioning and any changes it tries to implement.
3. Inner Setting—focuses on the organization’s internal environment, encompassing its characteristics, culture, and operational aspects, such as workflow and internal referral processes. Evaluating the Inner Setting means assessing how these internal factors affect activities and changes from within.
4. Characteristics of Individuals—focuses on the knowledge, beliefs, skills and self-efficacy of those involved in the implementation.
5. Process—encompasses the steps required for implementation, including planning, stakeholder engagement, execution, reflection, evaluation and adaptation.

The topic guide was pilot-tested and refined. It included open-ended questions and sample probes, but the interviewer was encouraged to explore emerging topics. The research team regularly discussed the process to ensure consistency.

One trained interviewer (AI) conducted interviews by phone, via Microsoft Teams for conference calls, or in person. The interviewer was not involved in patient care. Data collection continued until data saturation was reached within each stakeholder group (rheumatologists, physiotherapists, patients, and healthcare insurance representatives), when no new ideas emerged after three consecutive interviews.

All interviews were audiotaped in duplicate to prevent data loss due to technical issues and were transcribed verbatim. To preserve the integrity of the participants’ original, spontaneous accounts, audio recordings and transcripts were not provided to them for review or correction. Furthermore, all interviews were conducted by a single trained interviewer, which may have introduced interviewer bias; this risk was minimized by using a piloted CFIR-guided topic guide and by holding regular team debriefings to ensure consistent probing and interpretation across interviews.

### Data analysis

Verbatim transcripts of pseudonymized audio recordings were analyzed in Atlas.ti (v. 22). Interviews were analyzed jointly across diagnoses. At the design stage, we anticipated that the uptake of longstanding exercise therapy for RA and axSpA would occur under comparable organizational and financial conditions in the Dutch context (e.g. similar referral and reimbursement arrangements). During analysis, we therefore first coded all interviews together and subsequently compared codes and themes across RA- and axSpA-related accounts. A deductive framework was established using the five CFIR domains. Data within each domain were then coded openly to identify specific barriers and facilitators emerging from the participants’ narratives [21, 22]. Two authors (AI, AN) independently coded all transcripts, with a third resolving discrepancies to ensure coding consistency. Findings were then structured according to both the CFIR domains and the three key stages of therapy delivery:

Stage 1: Referral or direct access

Stage 2: Eligibility assessment

Stage 3: Treatment

### Ethical approval

This study was performed in accordance with the Declaration of Helsinki. The study protocol (ref: 2023-046) received an exemption from formal ethical approval from the Leiden University Medical Center’s Ethical Committee, as it was not required under Dutch law. All procedures complied with relevant EU and Dutch legislation, including the GDPR.

## Results

We conducted 18 semi-structured, in-depth interviews (2 by phone, 13 online, 3 in person) between December 2023 and June 2024. The interviews averaged 52 min (range: 41–76 min). Data saturation was reached with six patients, four rheumatologists, one physician assistant, five physiotherapists and two health insurance representatives (see Supplementary Table S1, available at *Rheumatology* Online). A comparison between RA and AxSpA did not show substantial diagnosis-specific differences in key determinants such as clarity of eligibility criteria, referral coordination, availability and training of physiotherapists or perceived financial uncertainties. Some nuanced differences were mentioned (e.g. existing group-based exercise options and patient organizations were mentioned somewhat more often in relation to axSpA), but these did not translate into distinct diagnosis-specific themes. We therefore present an integrated analysis across both conditions.

### Barriers and facilitators

Barriers and facilitators are reported per stage and organized by CFIR domain. Each stage section begins with a brief summary of the barriers and facilitators identified for that stage, followed by a detailed description structured by CFIR domain, with cross-references to Tables 1–3.

**Table 1:** Barriers and facilitators influencing the (self)referral to a longstanding exercise therapy intervention.

CFIR domain	Barrier (B) or facilitator (F)	Explanation of facilitators and barriers	Mentioned by the following stakeholder groups	Illustrative quotes
Intervention characteristics				
Evidence strength and quality + relative advantage	F	Strong evidence base supporting exercise therapy for RA/axSpA.	Rheumatologist	Rheumatologist: You practically have to drown rheumatologists in evidence! If you don’t, they’ll just say “it’s not evidence based” and won’t recommend it.
Complexity + design quality and packaging	B	Eligibility criteria for longstanding exercise therapy are lacking.	Rheumatologist	Rheumatologist: You should just have criteria. A criterion lists where if one of the five or if one is already positive or two positive whatever then you are eligible to receive the therapy.
Outer setting				
Patient needs and resources + cosmopolitanism	B	There is a lack of knowledge about the network of suitable physiotherapists, which creates reluctance to refer patients to physiotherapy.	Rheumatologist, patient	Rheumatologist: But also which physiotherapist is specialized in this and where such a person is located. That would be a real plus if the rheumatologist knew that.
External policies and incentives	B	Limited reimbursement for the patient for exercise therapy affects (self-)referral.	Rheumatologist, patient	Rheumatologist: Physiotherapy should be accessible to everyone, but unfortunately, the cost is a major barrier for many people. I often see patients who can’t afford it, and that’s a real shame. I believe everyone deserves to have it covered.
External policy and incentives	B	Concern that delays in guideline revisions (incorporating new research) lead to outdated referral protocols, causing insufficient and incorrect referrals.	Rheumatologist	Rheumatologist: Firstly, it should actually be included in the guidelines. There are guidelines for physiotherapy and exercise therapy in rheumatism. Those are tough processes, they take a long time.
External policy and incentives	B	Offering longstanding exercise therapy may increase demand, including from those who don’t need it.	Rheumatologist	Rheumatologist: But you have to weigh things carefully. Are you reaching the right people? Are you going to stir up a hornet’s nest? Will every rheumatoid arthritis patient suddenly think they have a blank check for physiotherapy? There are a lot of factors to consider here.
Patient needs and resources + cosmopolitanism	F	An insurer’s knowledge of available therapists and the treatment’s details directly influences their ability to inform clients.	Healthcare insurer	Health insurer: If an insured person has questions about exercise therapy, the customer service representative will help them locate the nearest physiotherapy practice.
Inner setting				
Available resources	B	Rheumatologists have limited time during consultations.	Rheumatologist	Rheumatologist: Because I don’t really have the time for that. I only have 10 min.
Networks and communications + structural characteristics	F	Implementation of standardized referral forms and processes.	Rheumatologist	Rheumatologist: We follow standardized referral protocols and recognize the value of exercise therapy for RA, based on research and clinical experience.
Networks and communications + readiness for implementation-available resources	F	Collaboration with nurses can make physiotherapy referrals more efficient.	Rheumatologist	Rheumatologist: Given my time constraints, I would probably involve the rheumatology nurse to explore the patient’s specific challenges in more depth.
Characteristics of individuals				
Knowledge and beliefs about the intervention	B	Rheumatologists or other healthcare providers lack knowledge about the benefits and evidence supporting exercise therapy.	Rheumatologist, physiotherapist, patients	Rheumatologist: Yes, but not exactly the content, and so it would be important for people to be informed about what it is exactly and what it entails, because if you don’t know about it, you won’t think to refer anyone.
Knowledge and beliefs about the intervention	F	Expectation management is important for the patient when introduced and referred to longstanding exercise therapy.	Rheumatologist, physiotherapist	Rheumatologist: Yes, patients need to understand what they’re getting into. The rheumatologist or nurse should provide clear information about the program and set realistic expectations.

**Table 2:** Barriers and facilitators influencing the eligibility assessment of a longstanding exercise therapy intervention.

CFIR domain	Barrier (B) or facilitator (F)	Explanation of facilitators and barriers	Mentioned by the following stakeholder groups	Illustrative quotes
Intervention characteristics				
Cost	B	Assessment costs are only reimbursed for eligible patients.	Physiotherapist, patients	Physiotherapist: Yes, well, a first consultation is covered by the patient’s insurance, but if we add an extra step like a screening, that needs to be paid for as well. The question is, how do we fund that?
Complexity	B	Lack of objective criteria to guide decisions about providing longstanding exercise therapy.	Physiotherapist, health insurers	Physiotherapist: And if you then have such a list of criteria, you will always have patients who then still think that, even if it just falls outside of it, that they actually still have a right to it. So it will always be a bit of a friction point there, I think.
Intervention source	F	Validated assessment tools that can accurately determine eligibility.	Physiotherapist, health insurer	Physiotherapist: Because what does “indication criteria” actually mean? When exactly is someone considered “severely functionally limited”? It needs to be clearly defined, otherwise, we’re just left guessing.
Outer setting				
Patient needs and resources	B	Balancing trained physiotherapy services with patient accessibility.	Physiotherapist, health insurers, patients	Health insurers: So, it’s a bit of a double-edged sword. On one hand, you want physiotherapists to be trained and have the right training, but on the other hand, they need to be accessible to patients.
Patient needs and resources	B	Tension between patient reimbursement desires and healthcare cost control.	Physiotherapists, health insurers	Physiotherapist: If physiotherapists are the only ones deciding, it could get complicated. People really want their therapy covered, so they might pressure the physiotherapist to approve it. Sometimes you might even agree out of goodwill, but that’s not how it should work.
Needs and resources of the organization	F	Standardized, professionally endorsed patient eligibility assessment guideline.	Physiotherapist	Physiotherapist: So the KNGF is now also working on revising the rheumatism guideline towards RA, in which they actually want to give a bit more guidance also for how that physiotherapist/exercise therapist can best do the indication process soon.
External policies and incentives	F	Policies mandating validated assessment tools for therapy eligibility.	Physiotherapist	Physiotherapist: It’s important to have clear guidelines for determining eligibility, rather than leaving it entirely up to the physiotherapists. Otherwise, patients might pressure them for approval, and sometimes they might give in, even if it’s not appropriate.
Inner setting				
Readiness for implementation—available resources	F	Assessment tools that integrate seamlessly into current practices.	Physiotherapist	Physiotherapist: Integrating this information into guidelines and training curricula would simplify the assessment process for healthcare providers.
Characteristics of individuals				
Knowledge and beliefs about the intervention	B	The patient’s wishes conflict with the eligibility criteria.	Physiotherapist	Physiotherapist: Their objective limitations are relatively minor, but they feel severely limited in their daily activities. There’s a disconnect between their perceived limitations and their actual functional capacity.
Complexity	B	Mismatch between visible and experienced symptoms complicates assessment.	Physiotherapist	Physiotherapist: Yes, you see, sometimes someone might have minimal joint limitations, but they still experience a significant disease burden.
Knowledge and beliefs about the intervention	F	Addressing patient knowledge and beliefs regarding their active role and shared responsibility in treatment.	Physiotherapist	I don’t know how best to encourage that. I often explain myself in the first consultation, like: 'Yes, you’ve come to me, but I expect [things/effort] from you, because I’m not going to solve it for you, but we’re going to embark on this path together.

**Table 3:** Barriers and facilitators influencing the treatment of longstanding exercise therapy intervention.

CFIR domain	Barrier (B) or facilitator (F)	Explanation of facilitators and barriers	Mentioned by the following stakeholder groups	Illustrative quotes
Intervention characteristics				
Evidence strength and quality + relative advantage	B	Lack of awareness among patients about the benefits of the intervention	Physiotherapist, patient	Physiotherapist: Yes, it’s important to properly educate patients. A therapist should explain the program and what it involves, essentially setting realistic expectations.
Cost	B	Costly specialist training can reduce the number of available trained therapists	Physiotherapist	Physiotherapist: Yes, you also have to pay for all the courses. And if you attend a conference, for example, you have to attend a conference every other year, you also have to pay for that. So yes, ultimately, there are quite a few costs involved.
Cost	B	Patients lacking the financial resources to cover the costs of exercise therapy.	Health insurer, patient	Health insurer: So, they might end up charging our clients, who’ll then send us the bill. But since it’s not covered by their plan, they’ll have to pay some of it themselves.
Relative advantage	F	Perceived benefits of the exercise therapy intervention compared with usual care.	Physiotherapist, patient	Physiotherapist: She’s been my patient for over a year now, and she hasn’t had to go to the hospital once during that time, which is fantastic. She even said, “This is the first time in years I haven’t been hospitalized because of my health.”
Adaptability	F	Patient-centred exercise therapy with a focus on realistic goals and dynamic adaptation.	Physiotherapist, patient	Physiotherapist: When working with this group, it’s essential to be flexible and adaptable. Set realistic goals based on their current condition, recognizing that their abilities will fluctuate.
Design quality and packaging	F	The necessary knowledge and skills to ensure safe and effective therapy.	Physiotherapist, patient, health insurer	Rheumatologist: In a perfect world, everyone would receive beneficial physiotherapy from a skilled therapist
Outer setting				
Patient needs and resources	B	Low-literacy individuals need extra support using information and technology.	Health insurer	Health insurer: This is essential because most people with chronic conditions have low levels of education. It’s a fact of life, and it means we have to make sure our services are designed to support them effectively. A lot of these folks aren’t tech-savvy or highly literate.
Patient needs and resources	B	Discrepancy between accessibility and quality of care.	Physiotherapist, patient	Patient: It’s all about how far I have to go. Ten min on my bike? No problem! But three hours in the car? Forget it! I’d rather see someone closer to home, even if they don’t have the exact same training.
Patient needs and resources	F	Patient demand drives therapist growth.	Health insurer	Health insurer: I’m hoping this really takes off. Imagine thousands of people getting the help they need, and more and more therapists getting trained! That way, everyone could find a specialist without having to travel too far.
Cosmopolitanism	F	Clear agreements about reimbursement for exercise therapy sessions by trained therapists.	Health insurer, physiotherapist	Health insurer: We prioritize quality by selectively contracting with practices whose therapists have completed specialized training. This ensures patients receive care from qualified providers.
Knowledge and beliefs about the intervention	F	Training and education for therapists on personalizing exercise therapy.	Physiotherapist, patient, health insurer	Physiotherapist: Yes, direct interaction is important. Therapists should have specific training in exercise therapy for rheumatic patients, such as courses offered by the NPI (formerly RAPID). This expertise enables them to provide effective guidance and support.
External policies and incentives	F	Monitoring and auditing ensure quality control and promote accountability.	Health insurer	Health insurer: There will be follow-up checks to ensure compliance with the established criteria and the therapist’s adherence to proper procedures. This starts with a simple check of the referral letter from the rheumatologist. If administrative checks raise any red flags, such as an unusually high number of clients from the same locality with a specific therapist, further investigation may be warranted. To maintain control and ensure accuracy, a file review could then be conducted.
Inner setting				
Available resources	B	The intervention requires a specific, dedicated area where the therapy can be conducted	Physiotherapist	Physiotherapist: Yeah, cause even if you did offer the therapy, you’d definitely need a space for people to actually do the exercises.
Available resources	F	Using group therapy while ensuring individual attention and personalization can help manage workload.	Physiotherapist	Physiotherapist: Yeah, things can get pretty hectic. But we have to find ways to make it work. Sometimes that means telling someone we can’t see them right away, or maybe they have to wait a few days or a week. It would be great to do more group sessions, though. That way we could help more people in less time.
Characteristics of individuals				
Self-efficacy	B	Patients may doubt their ability to adhere to longstanding exercise therapy.	Patient	Patient: Yeah, cause I really want that to stay, because I think it’s really great that that option is there. I think it’ll be good for a lot of people too. At least there’s something to do, even if it’s just one-on-one, but whether they’ll keep that up year after year is, uh, yeah, I don’t know. I think that’s harder then.
Self-efficacy	B	Patients may not understand or be motivated to do exercise.	Physiotherapist, rheumatologist patient	Physiotherapist: Yes, then it simply becomes routine, and not everyone has the discipline to consistently engage in home-based exercises nest to the therapy.
Knowledge and beliefs about the intervention	B	Lack of knowledge among physiotherapists about personalizing exercise therapy.	Patient	Patient: I prefer to train alone because it’s a personal process, and I value my privacy. While the physiotherapy was good, I found the group setting to be disruptive and I overheard personal information about other patients that made me uncomfortable.
Self-efficacy	F	Exercise builds confidence for therapy success.	Physiotherapist, patient	Patient: When I started exercising, I really noticed how much it helped me build resilience. It’s also been good for learning how to communicate about my condition with other people. It’s important to me that everyone, from close friends to acquaintances, can understand if they ask me about it. I’m definitely getting better at explaining things instead of just saying “you can see it, can’t you?” like I used to.
Knowledge and beliefs about the intervention	F	Knowledge among physiotherapists about personalized exercise therapy.	Physiotherapist, patient	Physiotherapist: Yes, I believe that’s essential. It’s crucial to stay informed about the latest developments and research on the most effective therapies, and personalized[…] so, yes, I think it’s necessary.

#### The (self-)referral

Patients and rheumatologists mentioned most barriers and facilitators related to (self-)referral to longstanding exercise therapy. Patients focused on their ability to self-refer, while rheumatologists addressed barriers and facilitators influencing the referral process. Key barriers included unclear eligibility criteria, difficulties locating qualified therapists and time constraints for physicians. Facilitators included physician awareness of the intervention’s effectiveness, patient education and managing expectations. Below, we provide a more detailed analysis of the barriers and facilitators using the CFIR constructs (Table 1).

##### Intervention characteristics

Rheumatologists all mentioned the absence of transparent eligibility criteria for the intervention, making it challenging to identify and refer eligible patients. However, they have recognized the strong evidence supporting the use of longstanding exercise therapy, which facilitates patient referrals. This highlights a conflict between the perceived evidence and the complexity of the new intervention.

##### Outer setting

Several outer setting barriers and facilitators impacted the referral process. In summary, both patients and rheumatologists cited financial uncertainty as a significant barrier, specifically highlighting limited reimbursement and unclear insurance coverage. Patients and referring rheumatologists both expressed difficulty in locating physiotherapists trained to deliver the specialized intervention. Rheumatologists noted this challenge was compounded by delays in updating clinical practice guidelines, which hindered the revision of official referral protocols. Rheumatologists also voiced broader concerns that promoting the therapy could lead to inappropriate demand from ineligible patients, potentially resulting in unnecessary treatment and increased societal costs. Conversely, health insurers explained that their awareness of available, trained physiotherapists allowed them to verify qualifications and better inform their clients.

##### Inner setting

In the inner setting domain, referral workflows and resource limitations negatively impacted the referral process for the intervention. Rheumatologists noted that the limited time available during patient consultations made it difficult to adequately inform and refer patients, as these tasks required more time than was available. However, they suggested implementing standardized forms and procedures within their organization or delegating these tasks to rheumatology nurse specialists could help streamline the referral process.

##### Characteristics of individuals

Patients, rheumatologists and physiotherapists mentioned a lack of awareness about the evidence supporting the intervention as a barrier. In addition, rheumatologists and physiotherapists emphasized that successful referrals could be supported by several factors, including clear communication about the intervention (with its benefits and the need for patient involvement), a thorough understanding of the supporting evidence and sensitivity to patient limitations and individual concerns.

#### Assessment of eligibility

Physiotherapists, health insurers and patients were the stakeholders who mentioned barriers and facilitators related to the eligibility assessment.

The issues they identified centred on the need for clear and objective eligibility criteria, effective communication with patients about the treatment and their eligibility, time constraints during the assessment process and the availability of transparent reimbursement information (Table 2).

##### Intervention characteristics

This domain explored the characteristics of the eligibility assessment for the longstanding exercise therapy. It addressed elements such as validated assessment methods, eligibility-dependent cost reimbursement and challenges resulting from the lack of objective eligibility criteria for the longstanding exercise intervention. Physiotherapists and health insurers indicated that a lack of objective eligibility criteria is a barrier to assessing a patient’s eligibility. Furthermore, patients and physiotherapists noted that unclear insurance coverage for eligibility assessments discourages patients from seeking necessary care, particularly because, if deemed ineligible, the assessment costs are not reimbursed and must be paid out-of-pocket. Importantly, a validated assessment tool and a therapy delivery protocol were seen as potential facilitators for the eligibility assessment.

##### Outer setting

According to the interviewees, factors from the outer setting influencing the eligibility assessment included the availability of longstanding exercise therapy. The outer setting provided a supportive system-level policy framework, including standardized eligibility criteria and required patient assessments. However, it also presented conflicting demands, notably the challenge of balancing specialized service provision with broad patient access and the difficulty of managing reimbursement levels adequately against the costs of delivering care. Physiotherapists, health insurers and patients all identified the challenge of balancing trained physiotherapy services with patient accessibility, including commute time and transportation, as a barrier. Additionally, physiotherapists and health insurers highlighted the competing demands of patient reimbursement expectations and the need to control healthcare costs as another barrier. On the other hand, physiotherapists noted that using a standardized, professionally endorsed guideline, along with a validated assessment tool, could serve as a facilitator for determining patient eligibility.

##### Inner setting

Only one facilitator related to the inner setting or organizational context was mentioned in the interviews: implementing guidelines with clear eligibility criteria. Physiotherapists noted that the assessment process is facilitated when therapy delivery guidelines are seamlessly incorporated into their daily workflow.

##### Characteristics of individuals

In the domain of characteristics of individuals, physiotherapists highlighted discrepancies between patient desires and eligibility criteria, the benefits of shared decision-making for patient adherence and the assessment challenges arising from inconsistencies in symptoms. They reported that the complexity of the patients’ conditions and the disparity between visible limitations and actual disease burdens experienced by the patients posed barriers to the eligibility assessment. Conversely, physiotherapists noted that addressing patients’ knowledge and beliefs regarding their active role and shared responsibility in treatment could facilitate identifying patients motivated to adhere to long-standing exercise programs.

#### Treatment

Patients, health insurers and physiotherapists mentioned barriers and facilitators related to the treatment. Key barriers and facilitators included knowledge gaps, logistical issues related to therapist availability, the potential for overutilization and patient knowledge deficits regarding therapy details (Table 3).

##### Intervention characteristics

Within the domain of intervention characteristics, specifically the treatment, several barriers and facilitators were identified during the interviews. Physiotherapists noted that the cost of training is a limitation to the availability of trained therapists for delivering the therapy. Patients and health insurers pointed to a lack of financial resources as a barrier to covering the costs of exercise therapy. Both physiotherapists and patients identified the perceived benefits of exercise therapy over usual care as a facilitator. Additionally, they highlighted that a patient-centred approach, focusing on realistic goals and allowing for flexible adjustments, serves as a facilitator. Finally, physiotherapists, patients and health insurers emphasized that the necessary knowledge and skills to ensure safe and effective treatment are also important facilitators.

##### Outer setting

The outer setting presented several barriers and facilitators to the delivery of treatment. Health insurers noted that individuals with low literacy who require additional support with information and technology present a significant barrier. Physiotherapists and patients highlighted the discrepancy between accessible care, defined by travel distance and transportation options, and the quality of care as another barrier. Health insurers noted that patient demand drives therapist growth, thereby facilitating the implementation of exercise therapy. Both health insurers and physiotherapists agreed that clear reimbursement agreements for longstanding exercise therapy sessions are important facilitators. Additionally, physiotherapists, patients and health insurers emphasized the importance of training and education for therapists on personalizing exercise therapy as another facilitator. Health insurers also noted that monitoring and auditing, which ensure quality control and promote accountability, further facilitate implementation.

##### Inner setting

Only physiotherapists reported barriers within the inner setting, mainly related to the availability of resources and organizational culture. In addition to a lack of dedicated space for exercise therapy, they highlighted that individual therapy appointments contributed to a challenging workload, which was seen as a barrier. However, they identified group sessions focusing on individual needs and personalization as a facilitator for effectively managing this workload.

##### Characteristics of individuals

In the domain of Characteristics of Individuals, several factors influencing treatment were reported in the interviews. Barriers mentioned included patient self-doubt about adherence, lack of patient understanding or motivation, insufficient therapist knowledge of personalization and a general lack of awareness among patients about the benefits of exercise therapy. Facilitators identified by patients and physiotherapists include increased confidence in therapy success, driven by the physiotherapists’ expertise in personalized exercise therapy.

#### Summary across the three delivery stages

In summary, a total of seven barriers and five facilitators were identified for the (self)referral stage, six and five for the eligibility assessment stage, and nine and ten for the treatment stage, respectively (as detailed in Tables 1, 2 and  3). Patients discussed adherence-related factors, rheumatologists focused on referral pathways, physiotherapists on eligibility and provision and insurers on use and coverage. Cross-cutting barriers included unclear eligibility criteria, limited access to trained physiotherapists, poor referral guidance and financial uncertainties. Common facilitators included strong evidence of effectiveness, structured communication and training for physiotherapists.

## Discussion

Despite evidence of its (cost-)effectiveness, endorsement by professional associations and policymakers and definite (RA) or provisional (axSpA) insurance coverage, the implementation of this (cost-)effective exercise therapy remains challenging. Our study shows that a complex interplay of factors influences its implementation. These include knowledge gaps among healthcare providers concerning therapy details and patient eligibility criteria, logistical challenges such as unclear referral pathways, limited availability of trained physiotherapists and difficulties in patient access to longstanding exercise therapy. Additionally, financial uncertainties related to therapy reimbursement further complicate the delivery of longstanding exercise therapy. For patients, these needs include awareness of the therapy, the ability to find a qualified practitioner and a clear understanding of their insurance coverage. Rheumatologists require clear referral pathways that specify which patients are eligible and identify the qualified physiotherapists to whom they can refer. Physiotherapists require clarity on eligibility criteria and treatment protocols. Health insurers need to verify which physiotherapists are qualified to deliver the therapy efficiently. Successful implementation depends on aligning these interconnected stakeholder needs.

While previous research has identified similar factors influencing the implementation of physiotherapy interventions, these studies have focused on isolated segments of care [23–28]. In contrast, our study adopted a holistic perspective that spanned the entire patient journey, from referral and assessment to treatment and thereby offers a broader system-level view than previous research. By integrating multi-stakeholder perspectives across the stages, our study adds to existing implementation literature focusing on single-setting or single-profession approaches.

This broader perspective reveals the importance of interdisciplinary coordination and organizational adaptation—elements often overlooked in more narrowly focused studies [23–30]. Our analysis showed that patient access is directly influenced by coordinated planning, defined roles and addressing logistical barriers, including referral pathways and the availability of qualified physiotherapists. Additionally, our study emphasizes the importance of examining the interconnectedness of factors within the CFIR domains [31]. For example, addressing outer setting challenges, like awareness of therapist networks, without addressing inner setting factors like referral workflows or provider confidence, is insufficient. Similarly, establishing eligibility criteria within the inner setting must be accompanied by attention to patient understanding and provider-patient communication to influence actual referral decisions. These cross-domain interactions are critical for effective implementation.

### Strengths and limitations

A key strength is its methodological rigor, which utilizes the CFIR framework for analysis and COREQ guidelines for reporting, thereby enhancing transparency and credibility. An additional strength is the inclusion of a wide range of perspectives by engaging diverse stakeholders across all stages of the care pathway. A third strength is the study’s multi-layered analysis, which structured findings by both CFIR domains and the three stages of care, revealing how implementation challenges vary across theoretical and practical contexts. However, our study has several limitations, including that the findings are specific to the Dutch healthcare context, where physiotherapy for these conditions is not typically covered by basic insurance and longstanding exercise therapy is largely delivered individually in first-line physiotherapy practices. Therefore, the barriers and facilitators identified, particularly those related to financial uncertainties, referral pathways and the organization of individually delivered first-line care, may not be directly generalizable to countries with different healthcare funding models or service delivery structures. In several other countries, for example, exercise therapy for axSpA is more commonly organized as group-based programmes in specialized settings, as reported by Rausch-Osthoff [23] and colleagues in Switzerland, rather than on an individual basis in primary care. At the same time, several determinants we identified—such as the need for clear and transparent eligibility criteria, coordinated referral processes and adequate training and support for physiotherapists—are likely relevant beyond the Netherlands. These factors reflect general implementation mechanisms rather than country-specific policies, and may therefore apply to other settings aiming to implement longstanding, structured exercise therapy for people with RA or axSpA. In addition, cultural factors may have influenced which barriers and facilitators patients and providers experience in relation to longstanding exercise therapy. Although we did not explicitly examine cultural influences, certain norms in the Dutch context—for example, expectations regarding self-management in chronic disease, attitudes towards supervised exercise and physiotherapy and how patients and professionals view the role of allied health in rheumatology care—are likely to have shaped how participants perceived and prioritized determinants. Because such cultural expectations and care traditions differ across countries, future research should examine their potential role in therapy uptake and consider how they might affect the transferability of our findings to other healthcare systems. Another limitation relates to the study’s sampling strategy. The small number of participants in some groups (e.g. insurers) may not capture the full range of views, and recruitment through professional organizations could have biased the sample towards more engaged patients. All interviews were conducted using mixed modes (in person, telephone, videoconference), which may have influenced participant comfort, disclosure and the depth of information shared; although we used a standardized topic guide and duplicate audio recordings, some residual mode effects cannot be ruled out. Furthermore, all interviews were conducted by a single trained interviewer, which may have introduced interviewer bias; this risk was minimized by using a piloted CFIR-guided topic guide and by holding regular team debriefings to ensure consistent probing and interpretation across interviews. Although we did not identify major diagnosis-specific differences in the main implementation mechanisms, some nuances emerged. For axial spondyloarthritis, stakeholders occasionally referred to existing group-based exercise options and local Bechterew (axSpA) associations more often than for RA, suggesting that in settings where such structures are more prominent, they could influence how knowledge about longstanding exercise therapy is disseminated and how easily programmes are embedded into existing care pathways.

### Future recommendations for implementation strategy

Based on our findings, we recommend targeted stakeholder actions across all stages of therapy delivery, including streamlining referrals, standardizing eligibility criteria and supporting treatment with therapist training and patient education (Table 4). A key financial barrier has been recently addressed, as national reimbursement for this therapy has been secured (for RA, effective January 2025; axSpA, effective January 2026). However, stakeholder awareness of this new coverage is lacking. Therefore, a critical next step for successful implementation is to communicate these essential reimbursement changes to clinicians, patients and physical therapists.

**Table 4:** Implementation strategy—suggested actions for all stakeholders involved across the key stages of exercise therapy delivery.

Referral
1. Improve rheumatologists’ understanding of the evidence and benefits of longstanding therapy
2. Establish a publicly accessible register to improve the visibility and accessibility of trained/qualified therapists
3. Introduce clear, objective and measurable referral criteria to remove subjectivity and social pressures from referral processes
4. Establish clear referral letters to increase the speed of referral and improve patient information access in the patient portal
Assessment
5. Establish specific, measurable, evidence-based indication criteria for consistent patient selection and efficient consultations
6. Implement clear cost and reimbursement guidelines for indication assessments and therapy to improve patient access and reduce financial uncertainty
Treatment
7. Provide physiotherapists with targeted training to ensure adequate knowledge transfer regarding therapy content and procedures
8. Establish a monitoring system for trained therapist availability to improve patient access and address workforce capacity issues
9. Monitor patient volume in longstanding exercise therapy programs to control for potential over-utilization
10. Implement concise patient information on longstanding exercise therapy, including content, benefits, expectations, personalization and eligibility criteria.

The importance of standardizing patient eligibility and referral for RA and axSpA treatment is underscored by recent guideline updates. The national guideline for RA was revised accordingly in late 2024, and the guideline for axSpA is now undergoing a similar revision.

Finally, we recommend further research to evaluate the proposed implementation strategy, guided by a suitable framework such as Proctor’s implementation outcome framework [32].

## Conclusion

Although longstanding exercise therapy significantly improves patients’ quality of life, is evidence-based and is reimbursed, its implementation in the Dutch setting remains challenging. The difficulty lies in the complex interaction between the diverse perspectives of stakeholders and the distinct stages of the healthcare pathway itself. This interplay creates both stage-specific obstacles and recurring issues driven by differing stakeholder interests. A coordinated, multi-stakeholder approach is therefore essential. Stakeholder-specific actionable recommendations to guide such efforts are summarized in Table 4.

## Supplementary Material

rkag013_Supplementary_Data

## Data Availability

The data that support the findings of this study are not publicly available due to participant privacy. **Box 1** Longstanding exercise therapy and the three key stages of implementation
**Longstanding exercise therapy**  Longstanding (52 weeks), personalized exercise therapy intervention for patients with RA and axSpA and severe functional disability. A personalized program, based on a comprehensive biopsychosocial assessment and collaboratively set treatment goals/plan, that combines active exercises (aerobic, strengthening, neuromotor) with patient education and self-management support. Delivered by trained/specialized physical therapists, the programme emphasizes progressive intensity adjustments, regular monitoring and evaluation by the physiotherapist and adaptation of the treatment goals/plan as needed. The therapy is intended to be ongoing, with regular sessions (duration 30 minutes) and consistent support to help patients manage their condition and improve their quality of life [9].
**Eligibility criteria for longstanding, personalized, supervised exercise therapy**  Eligibility criteria include [7]: A confirmed clinical diagnosis of RA or axSpA made by a rheumatologist The patient’s rheumatic disease activity is optimally controlled, regardless of medication use One or more severe daily functional limitations, as perceived by the patient, directly or indirectly linked to the rheumatic disease The functional limitations are attributable to the rheumatic disease The patient’s limitations cannot be addressed effectively with a short course of exercise therapy (less than 3 months)
**(Self-)referral to longstanding, personalized, supervised exercise therapy**  In the Netherlands, there are two primary pathways for a patient with RA or axSpA to access specialized exercise therapy. The first is a formal referral, where a rheumatologist or another physician directs the patient to a physiotherapist for appropriate assessment and treatment. The second is direct access (or self-referral), which allows patients to consult a physiotherapist without requiring a physician’s referral. An effective system for both pathways requires clear methods for patient identification and readily available information about the specialized therapy and the therapists who provide it.
**The assessment of eligibility for longstanding, personalized, supervised exercise therapy**  The physiotherapist determines a patient’s eligibility for longstanding, personalized, supervised exercise therapy by evaluating their overall health, medical history, physical condition and daily limitations. This comprehensive assessment ensures the treatment is both clinically effective and a responsible use of healthcare resources.
**Providing and adherence therapy longstanding, personalized, supervised exercise therapy**  Providing therapy refers to a qualified exercise/physiotherapist delivering a structured exercise program for the intervention. This includes professional training and education, techniques, protocol adherence, communication style and the ability to personalize according to the patient’s needs. Patient participation refers to the patient actively participating in and adhering to it. This includes motivation, perceived need, personal preferences, psychosocial context and practical considerations such as cost and distance to the therapist.
