# Supplementary material for: Barriers and facilitators to implement longstanding exercise therapy for people with rheumatoid arthritis or axial spondyloarthritis
Source: Rheumatol Adv Pract. 2026 Jan 29;10(1):rkag013. doi: 10.1093/rap/rkag013 (PMC12900539; doi:10.1093/rap/rkag013)
Supplement: rkag013_Supplementary_Data [file rkag013_supplementary_data.docx]

Supplementary Table S1: Table of characteristics of interviewed stakeholders

| Stakeholder group | n | Male n (%) | Average work experience treating RA/axSpA patients , years (range) | Average no. of RA and axSpA patients treated per week (range) | Participant L-EXTRA or L-EXSPA n (%) | RA n (%) |
| --- | --- | --- | --- | --- | --- | --- |
| Patients | 6 | 2 (40) | n/a | n/a | 3 (50) | 3 (50) |
| Rheumatologist | 4 | 2 (50) | 16 (112-22) | RA 22 (4-35); axSpA 5 278 (1-9) | 2 (50) | n/a |
| Physician assistant | 1 | 0 (0) | 22 (0) | RA 45 (7); axSpA 0 (0) | 0 (0) | n/a |
| Physiotherapist | 5 | 3 (60) | 12 (24) | RA 2 (1-2); axSpA 1 (0-1) | 2 (40) | n/a |
| Healthcare insurer | 2 | 2 (100) | n/a | n/a | n/a | n/a |
